# Efficacy and Safety of Topical Morphine: A Narrative Review

**DOI:** 10.3390/pharmaceutics14071499

**Published:** 2022-07-19

**Authors:** Krzysztof Nosek, Wojciech Leppert, Łukasz Puchała, Krzysztof Łoń

**Affiliations:** 1 Department of Pharmacology and Toxicology, Faculty of Medicine, University of Warmia and Mazury, 10-719 Olsztyn, Poland; hnnh@wp.pl (K.N.); lukasz.puchala@uwm.edu.pl (Ł.P.); lon.krzysztof@gmail.com (K.Ł.); 2 Chair of Palliative Medicine, Institute of Medical Sciences, Collegium Medicum, University of Zielona Góra, 65-417 Zielona Góra, Poland; 3 University Hospital of Heliodor Święcicki, Poznań University of Medical Sciences, 61-245 Poznań, Poland

**Keywords:** topical morphine, analgesia, adverse effects

## Abstract

Background. Opioids are the cornerstone of the therapy used in both acute and chronic pain syndromes to treat pain of moderate to severe intensity. The knowledge that opioid receptors also occur in other tissues outside the central nervous system has created a possibility for the topical use of opioids. Thus, local analgesia may be obtained without systemic adverse effects. Methods. A narrative review of scientific papers discussing the topical use of morphine was conducted. For this purpose, the PubMed, Google Scholar, Cochrane Library, and Mendeley databases were searched. Results. The current knowledge on topical morphine does not allow for its recommended use in everyday medical practice, but suggests it may be effective, particularly in the treatment of ulcers and erosions of inflammatory etiology and painful skin lesions including persistent post-mastectomy pain due to breast cancer. Conclusions. Topical morphine has its place beside other analgesics. An important issue is the practical possibility to meet the demand for topical formulations, which is limited by technical difficulties.

## 1. Introduction

Opioids are the cornerstone of the therapy used in both acute and chronic pain syndromes to treat pain of moderate to severe intensity. They act on three types of opioid receptors: µ, δ, and κ (MOR, DOR, and KOR). The particular receptor subtypes show differences in location, endogenous ligands, and the effects of their activation.

Opioid receptors are produced in the neurons of the posterior root ganglion and are transported to the posterior horn of the spinal cord and to the peripheral nerve endings. The process usually begins 1–2 days following tissue damage or the onset of inflammation. Another finding revealed that normal, unaffected tissues contain inactive, so-called silent opioid receptors, which are activated within minutes to hours after tissue injury. Their activation may be triggered by a number of factors, including alteration in tissue pH. As a result of tissue injury, the integrity of the perineurium is affected, which would otherwise constitute a barrier for such large molecules as those of opioids. Moreover, at the site of injury, proinflammatory cytokines are released, including IL-6, which additionally increase the tissue’s permeability to opioids [1].

At the same time, cells such as macrophages, lymphocytes, and mastocytes, which are found at the site of injury, contain endogenous opioids, as well as mRNA for proopiomelanocortin and proenkephalin. The majority of studies on the topical use of opioids have focused on morphine and, to a lesser extent, diamorphine and methadone [2,3].

Morphine, which derived its name from the Greek god of sleep, Morpheus, is a natural opium alkaloid which was isolated by Friedrich Sertürner in 1806 and first synthesized in 1952. According to the recommendations of the European Association for Palliative Care, it is an analgesic used to treat pain of severe intensity, and it is on the third step of the WHO analgesic ladder, as well as, in low doses, on the second step of the WHO analgesic ladder.

Morphine is a pure agonist of µ-opioid receptors and a weak agonist of κ- and δ-opioid receptors. It is metabolized in the liver, intestinal wall, kidneys, and CNS. The plasma half-life of morphine after its oral administration is 2–2.5 h, and after parenteral administration it is 1–1.5 h [4]. Morphine is a hydrophilic opioid. Its bioavailability following oral administration is approximately 14–64%. It is most commonly administered orally, but also by subcutaneous, intravenous, epidural, subarachnoid, or rectal routes. Depending on the preferred route of administration, morphine is used in patients with dysphagia, vomiting, digestive tract fistulas, and the need to obtain fast relief from severe pain.

The knowledge that opioid receptors also occur in other tissues outside the central nervous system (CNS) has created a possibility for the topical use of opioids. Thus, local analgesia may be obtained without systemic adverse effects, which allows for a reduced need for systemically administered analgesics.

There is ample evidence for the presence of opioid receptors and their agonists in various skin structures. They can be found in peripheral nerve fibers, keratinocytes, hair follicles, melanocytes, and the cells of the immune system [5]. Opioid receptors are G-protein coupled receptors (GCPR), which mediate the activity of not only endogenous opioid peptides such as enkephalins, endorphins, dynorphins, and endomorphins, but also those of exogenous opiate alkaloids such as morphine. All three types of opioid receptors, i.e., MOR, DOR, and KOR, can be found in the skin. However, the expression of opioid receptors in the skin and cells of the immune system is lower than their expression in the CNS [5].

The expression of MOR in brain tissue is approximately 200 times greater than in epidermal melanocytes, 1000 times greater than in epidermal keratinocytes, and exceeds its expression in fibroblasts by 20,000 times. DOR expression in brain tissue is about 200 times as high as in epidermal melanocytes, 1500 times greater than in dermal fibroblasts, and 2000 times greater than in keratinocytes. Human skin also shows mRNA expression in KOR receptors [6].

Moreover, cell cultures studies have indicated that ectoderm-derived cells (e.g., brain cells, keratinocytes, and melanocytes) express higher levels of MOR than DOR. By contrast, mesoderm-derived fibroblasts express higher levels of DOR than MOR [5].

Trauma and inflammatory processes in peripheral tissues lead to the increased synthesis and axonal transport of opioid receptors from dorsal root ganglia towards the peripheral sensory nerve endings. These phenomena are dependent on the electrical activity of neurons, cytokines, and nerve growth factor. Leukocytes infiltrating the inflamed tissue increase the regulation of mRNA coding for signal sequences of beta-endorphin and its processing enzymes [7]. At the same time, the inflamed tissue accumulates immune cells containing endogenous opioid peptides, which, through their action on opioid receptors, cause local analgesia [8]. A potent and clinically significant analgesia can be caused by opioids acting outside the CNS. This effect is seen particularly in painful inflammatory states, both in animals and humans. With morphine administered topically, it is crucial to know if the drug is metabolized in the skin.

In the liver, morphine is metabolized mainly to 3- and 6-morphine glucuronide by UDP glucuronosyltransferase UGT2B7 [9]. This enzyme, showing trace expression on an mRNA level in normal human skin fibroblasts and normal human keratinocytes [10,11], may also metabolize morphine following its topical administration. Additionally, other UGT1A1 enzymes may also be involved in morphine metabolism [12], as well as 1A3, 1A6, 1A8, 1A9, and 1A10. Moreover, the synthesis of normorphine by CYP3A4 and CYP2C8 must be taken into consideration [9].

To date, little is known about UGT2B7 expression in the skin. The expression of this enzyme in mRNA [10] in trace amounts in normal human keratinocytes, normal human dermal fibroblasts, human epidermis, and the Episkin™ skin model [13] shows that morphine can undergo biotransformation in the skin, but, most likely, to a lesser extent. Furthermore, morphine does not undergo the process of biotransformation in human cadaver skin obtained within 24–48 h after death [14]. It may be assumed that morphine administered locally undergoes glucuronidation only in trace amounts [15], and, as a result, its biotransformation has no major effect on the concentration of the drug at the site of its administration. The aim of the review is to assess the theoretical rationale for using topical drug formulations containing morphine salts as an alternative treatment of pain in patients who cannot be treated with morphine administered orally or parenterally.

## 2. Methods

A narrative review was conducted of scientific papers discussing the topical use of morphine. For this purpose, the PubMed, Google Scholar, Cochrane library, and Mendeley databases were searched using the following inclusion criteria: published studies testing the use of topical opioids, papers published in peer-reviewed journals, English or Polish language, human subjects, and no time selection.

## 3. Results

A total of 51 articles were found regarding topical morphine administration. The relevant studies conducted since 2010 are presented in Table 1.

## 4. Painful Skin Lesions

Pressure ulcers represent a serious problem in patients with advanced cancer. The areas which are particularly predisposed to developing pressure sores include trochanters, sacral regions, ischial tuberosities, heels, elbows, ears, and the occipital area [16]. Skin lesions are not infrequently sources of severe pain, and so their presence results in a deteriorated quality of life.

The results of the use of topical morphine in patients with painful cutaneous lesions were analyzed in a critical review in 2013 [27]. This included 27 observational studies, of which 6 were randomized, controlled trials, and 1 study assessed the bioavailability of morphine used directly on the wound. The authors, following a critical evaluation and review of the studies, found topically used opioids to be clinically useful and safe medications for pain control in cancer-induced ulcers and pressure sores. The effect of morphine was found to be markedly reduced in the case of vascular ulcers (venous or arterial). The doses of morphine varied between 1 and 15 mg and the surface of the wound was 30–40 cm^2^. Morphine was well tolerated, while its absorption from the wound was negligible and thus safe for the patient.

The doses differed significantly, with applications 2–4 times per day, depending on the demand for the drug from a particular patient. It seems that the differences in the doses could make it difficult to define a standard dose. There are conflicting reports on whether there is a dose–response relationship [27,28,29]. Other data point to delayed wound healing in patients receiving opioids (systemic and/or topical) [30]. This observational study, conducted by Shanmugam et al., enrolled 450 patients. The data was collected prospectively and included baseline characteristics, pain scores, longitudinal opioid exposures, and total wound surface areas. It was found that exposure to opioids commonly prescribed to patients with chronic wounds is associated with a reduced likelihood of wound healing [30]. However, no conclusive answer was presented as to the actual cause and effect relationship between exposure to opioids and delayed wound healing.

Ciałkowska–Rysz and Dzierżanowski [17] confirmed the efficacy of a 0.2% morphine gel (for pathological lesions affecting mucosa) and ointment (in the case of skin ulcers). The study was conducted among 35 palliative care patients. The drug was self-administered to the wound, with no restrictions concerning frequency of dosing. The morphine was an effective and safe drug, and the average pain intensity fell from 5.9 to 2.5 (on a numerical rating scale (NRS)) and did not exceed 4 (NRS). The effect lasted for the 28 days of the study. Two patients decided not to undergo a follow up after day 14 due to pruritus of moderate intensity.

Jyothi et al. conducted a study to compare the efficacy of morphine and loperamide, both delivered in an intrasite gel [18]. Loperamide is a mu receptor agonist showing no systemic absorption. The participants with healthy wounds and pain of >5 on the NRS were treated with an intrasite gel mixture (15 g intrasite gel mixed with 5 mL saline solution with either 10 mg of morphine or 10 mg loperamide tablets diluted, accordingly.) In this crossover study, morphine or loperamide gel—depending on the group assignment—was applied onto the wounds, with a 24 h follow up and a 1 day washout. This was followed by a change of the group, with another 24 follow up and a 1 day washout. In terms of pain relief, after 12 and 24 h, both loperamide and morphine showed significant reductions in NRS scores. However, there was no significant difference in mean NRS scores at 12 h and 24 h. Likewise, there was also no significant difference regarding the mean change in NRS between the two groups. According to the data collected, five patients preferred morphine, two patients preferred loperamide, and two declared both drugs to be equally efficacious.

Graham et al. concluded that the systemic absorption of opioids used topically is negligible and found these drugs clinically useful, with a lower efficacy shown in the case of vascular ulcers [27]. There is a need for an assessment and differential diagnosis of inflammation and infection, as the absence of symptoms indicates inflammation and, where there is no expression of the opioid receptors, it may result in a weaker analgesic effect of topically applied morphine.

## 5. Stomatitis

Stomatitis is one of the most common adverse effects observed in cancer patients undergoing chemo- and/or radiotherapy [31]. According to the WHO, up to 100% of patients can suffer from stomatitis after radiotherapy [32]. The intensity and size of the inflammatory lesions in the oral cavity depend on both the dose of radiation and the chemotherapy used. Chemotherapeutics which most commonly contribute to the development of inflammatory lesions in the oral cavity include fluorouracil, methotrexate, cisplatin, cyclophosphamide, and doxorubicin [33]. Additional factors which play a role in stomatitis are malnutrition, xerostomia, caries, neutropenia, and herpes simplex carrier status.

Inflammatory lesions are located primarily on the tongue and soft palate mucosa, as well as on the buccal and labial mucosa. They lead to dysphagia, difficulty masticating and swallowing food, severe pain, and, consequently, considerable weight loss, worsened quality of life, or even limited possibilities regarding cancer therapy (a reduction in the dose of cytostatics, radiotherapy, or delayed therapy) [34]. A recent approach assumes that, apart from topical analgesics and anti-inflammatory agents, a standard therapy involves patient-controlled, morphine-induced analgesia administered systemically [35]. A number of studies have also indicated that morphine sulphate may be used topically, in the form of a mouthwash, in order to diminish pain sensations and the severity of inflammation itself [19,20,21,35,36].

Over the years 2002–2003, the first attempts were made to use morphine in patients with stomatitis, and they proved that morphine sulphate in a 2% solution, in comparison with a 1% solution, is more effective in reducing pain sensations, with a similar incidence of adverse effects such as local sensation of burning/stinging/dry mouth [35,36]. The short duration of these adverse effects allowed the oral intake of food and fluid replacements.

Cerchietti et al. [35] proved that using 2% morphine allowed for significantly lower pain, and it shortened the period of severe pain duration by an average of 3.5 days in comparison with the so called ‘magic mouthwash’, which was a mixture of equal parts of lidocaine, diphenhydramine, and magnesium/aluminum hydroxide. None of the patients receiving morphine reported the need to use additional drugs from the third step of the analgesic ladder [29]. An interesting finding was that of a significant difference in the duration of serious functional disorders (*p* = 0.017). Five patients from the group not receiving morphine complained of local adverse effects, compared to just one patient in the group receiving this drug.

Vayne–Bossert et al. compared morphine with a placebo in the treatment of oral pain due to chemotherapy-induced and/or radiotherapy-induced mucositis, and they demonstrated that morphine induced prolonged analgesia [19]. Patients who, at the beginning of the trial, used a morphine solution to rinse their oral cavities and were later put on a placebo experienced greater pain relief than those patients who did not receive morphine. This effect may be explained by the high efficacy of the drug, which produced a strong placebo effect. Patients in a critical state, with odynophagia, trismus, and advanced stomatitis, who used a mouthwash with morphine as an adjunct to analgesic therapy in line with the WHO analgesic ladder, improved their quality of life and ensured good pain control [21]. In patients with trismus, a combined therapy allowed a greater mouth opening, even by 1.5 cm.

A study by Hemati et al. reported that the degree of inflammation severity (according to the clinical scale of the WHO (Table 2) was lower in the group of patients receiving a mouthwash with morphine (1.71 ± 0.6 vs. 2.46 ± 1.26) [20]. The patients receiving morphine also declared a greater satisfaction with the therapy. The reported adverse effects included a moderate burning sensation in the throat, itchiness while rinsing the mouth [20,21], and, less commonly, bleeding and dizziness [21]. Only a few patients reported local adverse effects due to using both solutions [35]. A small number of the study participants made it impossible to conduct proper randomization of the groups and to observe the minimal differences between them. Nevertheless, the morphine solution was noted to be significantly more effective in the reduction in the pain of patients with lesions in their oral cavities. There is a need to conduct a large-scale, double-blinded, randomized trial in order to confirm the conclusions from this study.

## 6. Postmastectomy Pain Syndrome

Oncological surgery comprises amputations which may lead to the development of persistent postoperative pain. It is usually associated with nerve injury, which leads to the formation of neuromas. Post-mastectomy pain syndrome (PMPS) is one of the most commonly seen postoperative pain syndromes. PMPS occurs in approximately 20–47% of women who undergo mastectomies [37,38,39,40]. The pain affects the upper part of the chest and an upper limb. It takes the form of neuropathic pain: it is stinging and burning, with episodes of twinges [41]. It is associated with intercostobrachial nerve injuries. The treatment of postoperative pain is difficult and, very often, ineffective. The risk of postoperative pain is, beyond doubt, connected with a lack of treatment or inadequate therapy during the perioperative period.

The treatment of neuropathic pain, according to recommendations from the Polish Association for the Study of Pain, relies mainly on the use of anticonvulsants (pregabalin or gabapentin), antidepressants (amitriptyline and duloxetine), and other adjuvant analgesics such as opioids, lignocaine, NMDA receptor antagonists, and cannabinoids [1]. At present, pregabalin seems a very promising drug; however, no regimen has been proven to adequately reduce the risk of postmastectomy pain syndrome [29,41]. Topically administered medications are a good way to improve the quality of postoperative pain control, as well as to reduce the probability of chronic pain occurring in the future.

Mohamed et al. combined morphine and bupivacaine, comparing their efficacy in relation to the dose of morphine [22]. In their study, 90 patients received 10 mL of 0.5% bupivacaine with 5, 10, or 15 mg of morphine, respectively, after a mastectomy. This solution, diluted with saline, was used to flush the operating field before closing the wound. A distinct advantage was noted in relieving pain in patients receiving the solution of 15 mg in comparison with patients receiving the solution with 5 mg and 10 mg morphine, both directly after the procedure as well as during the 3 months after surgery. The patients receiving 15 mg of morphine did not require additional analgesia during the first two days after the surgery. The average VAS scores did not differ between the groups during the first 2 h after the surgery, whereas after 2 h, the pain intensity was significantly lower in the group of women who received 15 mg of morphine. The early topical use of morphine not only ensured better pain control in the acute phase after the resection, but also decreased the incidence and intensity of chronic pain syndrome after the operation. None of the patients developed neuropathic pain (LANSS ≥ 12). The incidence of opioid-induced adverse effects such as nausea, vomiting, and pruritus was similar in the patients of all groups (Table 3) [22].

## 7. Oral Lichen Planus

Lichen planus is a disease associated with the appearance of characteristic papules on the area of skin and mucosa and, less commonly, on nails. The lesions cause a characteristic pruritus; patients tend to rub the skin rather than scratch it. It is usually a self-limiting disease and the lesions resolve within a year in 70% of the patients and within 2 years in 90% of the patients. A doctor’s role is to alleviate the symptoms [42]. Currently, the treatment of oral lichen planus (OLP) is two-modal: non-pharmacological (PUVA, photodynamic and laser therapies) and pharmacological (corticosteroids topically and orally, calcineurin inhibitors (cyclosporin/tacrolimus/pimecrolimus), retinoids, and dapsone) [42]. The treatment of OLP may also involve the use of mycophenolate mofetil, low doses of enoxaparin subcutaneously, and efalizumab [43]. The treatments pose difficulties and the symptoms often lower a patient’s quality of life, particularly if the affected sites are located within the oral cavity.

Zaslansky et al. tested the effectiveness of a solution of morphine in glycerin to treat local erosions and ulcerations in patients with oral lichen planus. A total of 45 patients were included in the study, of whom 43 completed it [23]. However, no differences were observed between the placebo and the 0.2% and 0.4% solutions of morphine, which were used three times per day for 5 days. The process of wound healing in all patients progressed at a similar pace and the patients reported a similar relief from the pain. This could be due to a natural course of the disease or the effect of glycerin’s properties [23].

## 8. Chronic Obstructive Pulmonary Disease

Chronic obstructive pulmonary disease (COPD) is characterized by persistent symptoms on the part of the respiratory system (dyspnea, cough, and sputum-productive cough), as well as a permanent restriction in air flow through the respiratory tract. In 80% of patients, it is caused by exposure to tobacco smoke. Lung damage occurs as a result of chronic inflammation of the respiratory tract, lung parenchyma, pulmonary vessels, and proteolysis. The treatment of COPD involves the use of long-lasting β2-mimetics, sometimes in combination with short-lasting β2-mimetics, and anticholinergic drugs; depending on the patient’s eosinophile count, inhaled corticosteroids and, sometimes, theophylline, roflumilast (PDE4 inhibitor), or mucolytics are used [44].

Systemically used opioids may effectively relieve dyspnea; however, they cause respiratory depression [45]. Nebulized morphine could then be an alternative which would bring relief to patients, while reducing the risk of adverse effects [24]. Morphine administered in nebulization acts in two ways: firstly, it decreases respiratory drive (systemic action), and secondly, it locally reduces dyspnea through stimulating opioid receptors in the bronchi, which inhibits the release of acetylcholine [46]. Consequently, it leads to a relaxation of the bronchial smooth muscles and decreased production of mucus in the respiratory tract [47]. The beneficial results and improvements in the quality of life in particular patients have been noted only in isolated case reports. So far, apart from one study in which the benefits were reported with weak-to-moderate certainty [25], the studies failed to confirm the efficacy of morphine when topically used [48,49,50]. However, the studies made use of jet nebulizers, which are known for their poor reliability. As it is generally known, only 10% of the dose reaches the lungs and there are considerable differences in the deposition of the drug between subsequent nebulizations [51].

Krajnik and Podolec demonstrated that pneumodosimetric methods may be useful in the inhalational administration of morphine in dyspnea and cough as they provide a high effectiveness of inhalation as a result of a maximal deposition of aerosol obtained in a particular part of the respiratory system. Moreover, they also limit drug emission into the environment, allow individualization of inhalation, and reduce the systemic effect of the drug as a result of decreasing aerosol deposition on the face, oral cavity mucosa, and eyeball surfaces [52].

Janowiak et al. assessed the efficacy of nebulized morphine, which was compared with 0.9% NaCl in patients with severe COPD [26]. This randomized study demonstrated that inhaling morphine in a dose of 3–5 mg decreases apnea by over 20 mm in a visual analogue scale (VAS), with minimal adverse effects, and that the improvement lasts for at least 24 h after one dose. No significant difference was observed in Wilcock’s test, which correlates with FVC, which may be explained by a lack of clinically significant changes in the static spirometry results. The scientists themselves attributed the positive results of the test to using a better method of nebulization, aPNEUMONEB equipped with a BCTS–S head, and to using mass median aerodynamic diameter (MMAD), which is effective in depositing drug doses in the large airway (3.1–4.9 µm). The nebulizer used in the study analyzed the respiratory pattern, delivering an aerosol bolus in the 3/4 of inspiration in order to minimize the drug release into the environment and onto the internal surface of the device. There are reports that this way of administration may increase the drug deposition in the lungs by as much as 60% [26]. The positive effect used in the test was most likely due to the action of the morphine onto opioid receptors, which are found in the epithelium of the trachea and large bronchi. The only limitation to the study was the bitter taste of the nebulized morphine, which hindered effective blinding during the study.

## 9. The Use of Topical Morphine in Children

Pain occurring in children is an issue of great importance and requires, in much the same way as in adults, a multi-specialist approach. Experiencing pain at an early stage of life may lead to long-term consequences. According to the WHO, the treatment of pain in children is one of the major issues of public health worldwide. Considering its pathophysiology, pain in children may be defined as receptor, neuropathic, or multi-component pain. The treatment of pain in children poses difficulties, which can be attributed to a number of factors, adding to its complexity. Conducting long-term, randomized trials in a population of children with non-cancer pain poses difficulties.

Cooper et al. presented all facts reflecting the state of knowledge on the external use of morphine in children and adolescents [53]. Out of all the papers retrieved from medical databases (4037), the authors initially selected 12, but later came to reject all of them as they failed to meet the criteria of being valuable, reliable publications (of the 12 papers, 8 studies were conducted on adult populations, and 3 papers had wrong assumptions).

Watterson et al. successfully used gels with morphine sulphate in two children with epidermolysis bullosa at a dose of 0.2 mg morphine/kg body weight [54]. In both patients, a long-lasting relief from pain was noted (up to 48 h) of 40–66% (as assessed by a patient on a VAS), depending on the site, and faster wound healing of gel-treated sites, with a visible improvement after 4 weeks of use, was also noted. No adverse effects were observed. Since 2004, no papers on the use of topical morphine in children and adolescents have been published.

## 10. Conclusions

In 1986, the WHO developed its analgesic ladder, which, over the years, has been subject to some modifications. However, topically used morphine has not been considered on any step of the analgesic ladder. This may be due to the limited number of studies on the topical use of morphine and the small size of the trial groups (which do not generate statistically significant results), which may translate into a risk of error in particular studies [55]. Reliable estimates regarding clinical efficacy may only come from large trials or from combining a number of small-scale studies.

Despite its limited use topical morphine has its place beside other analgesics [56]. The current knowledge on topical morphine does not allow for its recommended use in everyday clinical practice, but it suggests that it may be effective, particularly in the treatment of ulcers and erosions of inflammatory etiology [27], painful skin lesions [17,27], mouth lesions [19,20,21,35,36], and, in women, after a mastectomy due to breast cancer [22]. Additionally, in a study conducted by Polish scientists involving the use of pneumodosimetric methods, the inhalation of morphine was demonstrated to decrease dyspnea in patients with severe COPD [26]. There are still no large-scale, reliable trials on its use in children and adolescents [53].

The above-quoted data coming from the literature of recent years show there is still a clinical need for novel methods to treat atypical pain. Despite a wide range of oral and intravenous analgesics, topical morphine seems an attractive alternative owing to its potentially high efficacy associated with a significantly reduced risk of adverse effects. In the studies referred to above, the adverse effects were marginal or did not occur at all. Recent reports point to a high efficacy of extemporaneous formulations for topical use containing morphine salts.

A lack of registration of topical morphine formulations (off-label) may limit its use by medical staff. Additionally, the absence of clear indications and the need for appropriate equipment and qualified staff to prepare extemporaneous drugs represents a major obstacle. Both the limited microbiological stability of the product and the difficulty in defining the right dose diminish the role of such formulations.

Despite their obvious disadvantages, magistral formulas are an interesting alternative in palliative care or surgery, and they offer the possibility of individualized decisions about formulations. Such personalized therapy is of particular importance in the management of less common conditions or in patients who do not tolerate standard medical products. An important issue is the practical possibility of meeting the demand for such formulations, which is limited by technical difficulties. An assessment of their actual availability should precede a therapeutic decision.

## Figures and Tables

**Table 1:** Studies conducted since 2010 on morphine administered topically.

First Author, Reference, Year of Publication	The Title of the Study	N	Study Design
Painful skin lesions			
Łapot [16] 2016	Topical use of morphine in palliative care patients—a report of two cases	2	Description of two cases reporting topical use of morphine
Ciałkowska–Rysz [17] 2019	Topical morphine for treatment of cancer-related painful mucosal and cutaneous lesions: a double–blind, placebo–controlled cross–over clinical trial	35	Fourteen day evaluation of analgesic effect of 0.2% hydrogel/ointment on painful mucosal and cutaneous lesions in palliative care patients, with a 28 day follow up
Jyothi [18] 2021	Morphine versus loperamide with intrasite gel in the treatment of painful dermal ulcers: a randomized crossover study	12	Comparison of topically applied with intrasite gel loperamide and morphine in treating pain for 24 h followed by a day washout and crossover in another group for 24 h
Stomatitis			
Vayne–Bossert [19] 2010	Effect of topical morphine (mouthwash) on oral pain due to chemotherapy—and/or radiotherapy–induced mucositis: A randomized double–blinded study	9	Comparison of efficacy of 2% morphine solution and placebo (quinine diHCl solution)
Hemati [20] 2015	Morphine mouthwash for the management of oral mucositis in patients with head and neck cancer	30	Comparison of 2% morphine solution with magic mouthwash
Saroja [21] 2010	Oral morphine solution as an oral rinse or mouth gargle for mucositis pain	10	Evaluation of efficacy of 3% morphine solution
Postmastectomy pain syndrome (PMPS)			
Mohamed [22] 2016	Effect of topical morphine on acute and chronic postmastectomy pain: What is the optimum dose?	90	Evaluation of efficacy of solutions: 10 mL 0.5% bupivacaine plus 5 mg/10 mg/15 mg morphine in postoperative pain and the occurrence of chronic PMPS
Oral lichen planus			
Zaslansky [23] 2018	Topical application of morphine for wound healing and analgesia in patients with oral lichen planus: a randomized, double–blind, placebo-controlled study	43	Investigation of effect of 0.4% morphine solution on erosions and ulcers in course of lichen planus of oral cavity
Chronic obstructive pulmonary disease (COPD)			
Ekström [24] 2015	Effects of opioids on breathlessness and exercise capacity in chronic obstructive pulmonary disease: A systematic review	271	Meta-analysis of all papers published before 8 September 2014, using Cochrane methodology, on use of opioids in alleviating dyspnea and improving exercise capacity in COPD
Shohrati [25] 2012	Effect of nebulized morphine on dyspnea of mustard gas–exposed patients: A double–blind randomized clinical trial study	40	Assessment of efficacy of nebulized morphine (once daily for 5 days) in patients with COPD following exposure to mustard gas
Janowiak [26] 2017	Dosimetrically administered nebulized morphine for breathlessness in very severe chronic obstructive pulmonary disease: A randomized, controlled trial	10	Assessment of reduction in breathlessness in patients with COPD with nebulized morphine

N: number of patients.

**Table 2:** Clinical scale for the assessment of inflammation severity by the WHO [20].

0	Inflammation cured, physiological state
1	Mild pain, not interfering with eating
2	Painful erythema, swelling, ulcers; the patient cannot eat
3	Severe, painful erythema, swelling and/or ulcers; the patient has difficulty eating
4	Required parenteral or enteral support

**Table 3:** Adverse effects among the 90 patients, depending on the morphine dose used [22].

	Doses of Morphine		
Adverse Effects	Morphine 5 mg	Morphine 10 mg	Morphine 15 mg
Nausea (*p* = 0.902)	10%	13.3%	13.3%
Vomiting (*p* = 0.787)	13.3%	16.7%	20%
Pruritus (*p* = 0.770)	3.3%	6.6%	6.6%
